# How do ferry operators communicate their engagement with sustainability issues? Comparison of the Baltic and the Adriatic ferry operators

**DOI:** 10.1371/journal.pone.0338904

**Published:** 2026-01-20

**Authors:** Natalia Wagner, Ewa Hącia, Aleksandra Łapko, Roma Strulak-Wójcikiewicz, Gorana Jelić Mrčelić, Eli Marušić

**Affiliations:** 1 Faculty of Economics and Transport Engineering, Maritime University of Szczecin, Szczecin, Poland; 2 Faculty of Maritime Studies, University of Split, Split, Croatia; University of Messina, ITALY

## Abstract

A successful transition toward sustainable shipping requires continuous measurement of progress and transparent communication of results. The aim of this paper is to evaluate and compare the attitude to sustainability reporting applied by ferry operators in the Baltic and the Adriatic Seas in the context of sustainability issues relevant to the ferry shipping market. The research framework combines two phases of empirical research – (1) expert evaluation and (2) website content analysis to assess whether ferry operators share information about their engagement in corporate sustainability in relation to the examined sustainability categories. It is found that the operators functioning in the Baltic area showed much greater engagement in informing about sustainability issues compared to those operating in the Adriatic area, even though in both areas the predominant level of engagement was very low. Moreover, the Baltic operators usually concerned different activities than those communicated by the Adriatic operators.

## 1. Introduction

Running a business in the spirit of sustainability is one of the most material challenges faced by companies across various industries, including the shipping industry [1–3]. Implementation of the corporate sustainability concept is a truly challenging task that requires thorough redesigning of business models and following a long-term sustainability strategy. Nevertheless, this is something possible to achieve [4]. The starting point is the right attitude taken by enterprises, appreciation of the significance of sustainability issues in everyday decisions and long-term strategies, and clear communication with stakeholders.

Challenges of this type are faced by, among others, the global ferry industry which, after a period of slowdown caused by the COVID-19 pandemic, is now considered to be a bustling segment of the shipping market, projected to grow in the years to come [5]. In 2019, ferries transported approximately 4.27 billion passengers and 373 million vehicles. In comparison, the world’s airlines carried 4.5 billion passengers in the same year [6]. Unfortunately, despite the significance of ferry markets for the international economy, the attitude of shipowners to sustainable development may raise some doubts. This is evidenced, e.g., by the large share of CO_2_ emissions in this segment, compared to the size of the Ro-Pax ferries fleet, which are ferries designed to carry both vehicles and passengers. They are commonly used in regional transport systems. The term “Ro-Pax” is a combination of “Ro-Ro” (roll-on/roll-off) and “Pax” (passengers). In Europe, the share of Ro-Pax ferries in the total fleet amounted to 3%, while the CO_2_ volume emitted by this vessel segment accounted for as much as 10% [7]. Ro-Pax ferries are significant emitters of GHG on European waters. In 2023, Baltic Sea shipping emissions totaled 15.2 million tons of CO₂, with Ro-Pax ferries contributing the largest share at 23.7% of the total [8]. At the same time, the Baltic Sea and the Adriatic Sea, as semi-enclosed seas with shallow coastal areas, are the most vulnerable marine areas to climate change in Europe [9].

The object of our study comprises ferry companies that operate international ferry lines across the Adriatic and the Baltic Seas. Our study includes 20 ferry operators serving the Baltic routes, who operate a total of 218 ferries, and 12 operators serving the Adriatic routes with 155 vessels. Ferry services in the two seas show diverse characteristics. Compared to the ferry lines operating in the Baltic Sea, the ones serving the Adriatic ports are more focused on handling the tourist traffic. In the Adriatic region, the summer season is dominated by fast, short-distance ferry services between the islands, as well as sightseeing cruises, extremely popular among tourists looking for day trips or fast transport between the islands and the mainland. Therefore, short-distance tourist traffic in the Adriatic is dominated by catamarans or small ferries, while Ro-Pax ferries serve longer, international routes. In the Baltic Sea, most ferries operate long-distance international routes (e.g., Poland and Sweden), where it is necessary to transport goods and passengers throughout the year rather than just seasonally. Therefore, Ro-Pax vessels constitute a better transport solution due to the demand for all-season transport, greater loading capacity and stability in adverse weather conditions [10–12].

The Baltic is one of the main ferry markets in the world [13], and Ro-Pax ferries handle both passenger and freight transport – cars, trucks, trailers, heavy cargo, sometimes railway wagons – [14]. Long distance ferries provide overnight sleeping accommodation. The clients include individual tourists and travel agencies on the one hand, and freight forwarders and carriers on the other [15]. They represent both B2B and B2C markets. As the passenger and freight traffic is handled by the same shipowners using the same vessels, information shared by them about their activities, including sustainability disclosures, is addressed to different types of audience. The ferry market in the Adriatic Sea, while also handling passenger and freight traffic, exhibits a slightly different character compared to the Baltic Sea. In contrast to the Baltic, where all-year-round freight traffic is highly dominant, the Adriatic market is highly seasonal, particularly in Croatia, where traffic to numerous islands (e.g., Hvar, Brač, Korčula) peaks during the summer. This seasonality necessitates that operators maintain a diversified fleet structure to serve both short-haul local routes and long-distance international services (often overnight crossings). Complementing the conventional ro-pax ferries (used on long-haul routes), high-speed catamarans are commonly deployed on short and seasonal connections, frequently carrying only passengers and bicycles. This fleet structure represents a key distinguishing feature when compared to the dominance of large Ro-Pax vessels in the Baltic Sea.

The analysis covered ferry companies operating in the Baltic Sea and the Adriatic Sea. Our research is a contribution to the research field of sustainability communication. The issue of sustainability communication in the shipping market is addressed in the literature in several dimensions. The increasing need for publishing sustainability reports and developing sectoral standards for shipping markets [16–17] is noted, as well as the necessity of identifying material issues for the various maritime segments and the need for transparent cooperation with various stakeholder groups [18–19]. The identification of UN Sustainable Development Goals to which shipping may contribute is increasingly being addressed, with container operators and cruise operators being studied in particular [20–22]. The need to communicate about business models and technologies used to achieve carbon neutrality as a long-term goal is also emphasised [23–24].

At the moment, the literature lacks a comparative analysis of sustainability communications applied by ferry operators handling some of the most active marine areas in Europe in terms of ferry traffic. This task is far from easy, because issuance of formal ESG/sustainability reports is not a common practice, the information published is very diverse and dispersed, it tends to be disclosed according to the shipowners’ own standards, and therefore it is difficult to compare. In the EU, this situation is bound to change due to the Corporate Sustainability Reporting Directive (CSRD) in force since 2023, which provides for a gradual roll-out of the obligation to publish sustainability reports [25]. The obligation will be imposed gradually on subsequent groups of companies, starting with the largest and publicly listed companies. Even though ferry operators do not belong to that group, the new regulations will impose the need for them to measure their own GHG (greenhouse gas) emissions and share this information with their customers. As transport services providers, they are integrated into their customers’ value chains, and their transport operations contribute to the carbon footprint made by final products and services. The solutions proposed in the CSRD directive will in the long run contribute to the situation where companies covered by the reporting obligation will choose services of transport operators with the lowest possible GHG emissions. Therefore, ferry companies should be interested in rationalising their own activities in this regard and in providing that information to their current and potential customers.

This study is part of a wider research project on sustainability communication by ferry operators in selected water areas in Europe. In the previous studies, a methodology was developed to rank sustainability topics that are vital to ferry operators, and it was applied to ferry operators functioning in the Baltic Sea [26]. This study deepens and expands the knowledge gained on the subject. The research applied an original index which makes it possible to assess each company individually, and also to run a combined comparison of ferry operators functioning in the two examined marine areas. In addition to that, the managerial implications of the research findings have been expanded to include links with the regulations resulting from the new CSRD directive.

The aim of this paper is to evaluate and compare the attitude to sustainability reporting applied by ferry operators in the Baltic and the Adriatic Seas in the context of sustainability issues relevant to the ferry shipping market. To achieve this goal, a research framework was used that combined the results of expert research indicating the importance of sustainability issues at a ferry segment in general and an assessment of corporate sustainability communications presented on the websites of ferry operators and in their sustainability reports.

The research study, the results of which are presented in this article, is innovative in nature. For the first time, a comparison was made between ferry operators functioning in two different marine areas, focusing on their engagement in sustainability communication. In addition to that, it is worth noting that the research study was carried out using the proprietary research framework. The research framework applied combines two phases of empirical research. For each of them, a suitable research method was chosen. In the first phase, expert evaluation was applied to determine the most important sustainability categories that are vital for the activities of ferry operators, taking into account the specific nature of their operations. The second phase involved the use of website content analysis to assess whether – and to what extent – ferry operators shared information about their engagement in corporate sustainability in relation to the examined sustainability categories. Next, the original index (combining the results of both phases) was calculated for all the examined operators, the results were discussed, and the conclusions were developed.

The following research questions were formulated:

RQ1. Which of the surveyed operators were the most engaged in sharing information about their sustainability-related activities?

RQ2. What are the similarities and differences in the approach shown by ferry operators in the Baltic and the Adriatic towards sharing information about sustainability issues?

The study is based on theoretical concepts and methodologies developed in several research areas, i.e., corporate sustainability management, sustainability reporting, and maritime transport management. Corporate sustainability approach, in this article equated to the strategic CSR concept, made it possible to identify a number of criteria important in assessing implementation of sustainability concepts by the company, taking into account the main groups of stakeholders. The adopted perspective is consistent with the stakeholder theory which emphasises the need for any organisation to strengthen its relationship with its stakeholders, meet their expectations regarding CSR strategy development and CSR disclosure practices. Then, using the sustainability reporting standards, it was verified which non-financial information was disclosed on the websites and in the sustainability reports submitted by the ferry operators to their stakeholders. Maritime transport management as a research area made it possible to select research objects that had not yet been compared in the literature in such a way, taking into account the specific nature of their functioning and the customers’ expectations.

This article is organised as follows: the literature background in Section 2 presents the role of strategic corporate sustainability and explains the process of developing the categories used to assess the sustainability attitude taken by the ferry operators examined in this study. The research method and the study objects are described in Section 3. Section 4 presents the results and compares the ferry companies operating in both seas in question. Section 5 concludes the research and suggests avenues for future research exploration.

## 2. The literature background

### 2.1. Strategic corporate sustainability in the shipping industry

Nowadays, corporate sustainability is considered one of the most important megatrends in strategic business management [27]. In both academic research and business practice, this concept is often identified with the concept of Corporate Social Responsibility (CSR) [28]. Also in our paper these terms are used interchangeably. Both concepts, despite having different roots, are now understood as combining the environmental, social and business perspectives to form a unity, and integrating it into the core of a company’s strategy [29]. It is emphasised that CSR is a complex, multidimensional concept and its understanding has changed strongly over time. According to the current definition, CSR is “corporate responsibility for its impact on society” [30]. The concept of strategic CSR which involves embedding a holistic corporate sustainability perspective into a company’s strategic planning, core activities, and all operations, is of great importance for business entities [31]. Incorporating CSR into a company’s business strategy can result in advantages like enhanced financial performance, stronger stakeholder relationships, and new areas for innovation development [32]. Theory and empirical research confirm the existence of positive relationships between CSR activities and the financial performance of enterprises [33]. The scale of the positive impact of CSR practices on corporate income is examined in various aspects, e.g., depending on the industry, kind of ownership, capital or labour intensity of the enterprise [34]. Positive results are achieved through improved brand reputation, stakeholder engagement, risk mitigation, and increased innovation potential [35]. What matters is that not only implementation of sustainable activities is important, but also spreading the word about the fact. Effective communication with stakeholders is crucial for leveraging the efforts and translating them into tangible business benefits.

As in the general discourse, so in the shipping industry, CSR is increasingly seen as an important part of a business strategy. In the literature focused on the shipping industry, the role of CSR tends to be presented in two ways. On the one hand, CSR activities are described as a marketing tool to promote a company’s services and image in the shipping market [36]. On the other hand, recent research studies increasingly perceive the role of CSR more deeply, in line with the spirit of sustainability, as the core of a long-term business strategy inextricably linked to many aspects of its operations, including its fleet development [37]. As the literature has demonstrated a positive relationship between sustainability disclosure and financial performance of publicly listed shipping companies, it is important to emphasise the need for a proper selection of a competitive strategy in the market and adoption of long-term corporate sustainability business models, regardless of short-term financial results [38–39].

The study of enterprises’ activities in the context of corporate sustainability and their impact on the enterprises’ financial performance is possible due to the sustainability reporting practice which is increasingly popular among international corporations and publicly listed companies. Financial markets, investors and other stakeholders expect businesses to be transparent in the three areas: Environment, Social and Governance (ESG). The aim of ESG is to make corporate sustainability activities measurable [40]. The need to inform the community about certain, non-financial aspects of a company’s operations is emphasised by the stakeholder theory. It explains the relationship between the need for CSR practices and disclosure of its results on the one hand, and the strategic management of the organisation on the other [41]. It emphasises that the CSR disclosure motivation of an organisation is driven by the desire to manage its stakeholders [42] including customers, employees, suppliers, and the community [43]. The literature shows that stakeholders are putting pressure on shipping companies to adopt sustainable shipping practices more widely [44]. The ferry operators analysed in this study have a very diverse range of external stakeholders, much more diverse than in the case of freight shipping. There are several main types of customers – individual tourists, travel agencies, but also road transport companies and drivers, which means that both sustainability strategies and communications in this respect must be addressed to each of these groups.

In every industry, including the shipping industry, it is very important to apply strategic CSR-brand fit which combines a company’s CSR initiatives and the brand’s identity or image [45]. An expanding body of research indicates that companies that successfully establish a strong CSR-brand fit can enhance consumers’ intentions to purchase and repurchase [46]. This is also a very important issue for the maritime industry. Proper integration of key CSR activities with the business strategy of ferry operators and developing a coherent CSR strategy is one of the major challenges facing the modern maritime business. Taking it a step further, to measure and evaluate the strategy implementation, it is necessary to select measures and indicators that will adequately reflect the specificity of the company, and fit into the nature of its market and the stakeholders’ expectations.

Shipping companies more and more often spot the need to communicate their understanding of the responsibility for the marine environment and to disclose their own activities taken in the spirit of the CSR and corporate sustainability concept. The shipping market also demonstrates examples of engaging in implementation of UN Sustainable Development Goals (SDGs) and informing the public about its attitudes and actions [20–22]. Di Vaio et al. [16] positively assessed the efforts of leaders in the cruise and container ships market toward clear communications aimed at sustainability disclosures, especially in the area of environmental impacts. However, the shipping industry is still perceived to have a lower level of CSR maturity in comparison with other sectors [17].

The increasing openness with regard to sharing information about activities completed so far and ambitious plans can be seen in the sustainability reports published by some shipowners, mentions on corporate websites, social media posts and in other marketing communications. However, the question arises whether this positive trend concerns the entire shipping market, or only its selected segments or even individual shipowners. Is it possible to talk about full openness of shipowners,or only about simulated activities aimed at creating a favourable image of the company while making minor changes to the rules of conducting the shipping business? Researchers are trying to answer these questions. A small but growing number of research studies address the issue of disclosing information on sustainability in the shipping sector, focusing primarily on container and cruise lines [16,20]. Research is conducted in different directions. It is argued that well-chosen management practices, greater industry openness to new technological solutions, as well as widely understood education – both among the crew and on land – can contribute to the implementation of sustainable solutions [47–50]. Research studies focusing on sustainability disclosures in the shipping market also use new data sources – social media (Twitter and Facebook) [51]. In general, when a company website is linked to its social media profiles, it enhances the effectiveness of content distribution across a broader audience [52]. A well-informed society is essential for driving sustainability transformations [53]. Therefore, acting sustainably and effectively communicating it to stakeholders can help raise consumer awareness, promote the concept, and foster positive change.

### 2.2 Measuring the sustainability performance in the shipping industry

A successful transition toward sustainable shipping requires continuous measurement of progress and transparent communication of results. The use of indices to assess emissions standards and energy performance has its history. Environmental indices, computed for individual vessels, are used to assess the current state and progress of engagement in the green transformation of the maritime industry. The computed indices include those developed by the regulator in the maritime industry, i.e., International Maritime Organisation (IMO) as well as by independent institutions [54]. Among the latter, the most renowned are the Clean Shipping Index (CSI), ESI (Environmental Ship Index), and the Green Award. They are calculated based on the characteristics of individual ships, taking into account selected types of NO_x_, SO_x_, CO_2_, and other pollutants emissions. In some ports, eco-certified vessels are granted discounts on port charges [55]. Initiatives promoting eco-friendly shipping via eco-labelling have been disseminated for many years, are recognised in business practice, and analysed in the literature [56]. The positive values of the indices obtained for individual ships can then be used to assess the shipowner in terms of the eco-friendly strategy applied. The use of indices of this type to assess the shipowners’ engagement in development of sustainable shipping has a significant drawback – it only takes into account emissions and energy performance, i.e., natural environment protection issues. Such a perspective is insufficient to assess a shipowner’s strategy in terms of contributing to strengthening the sustainable shipping. The reason for this is that it does not take into account many other important issues related to sustainable management of a ship company, such as social and governance issues.

These issues are being addressed by a new research trend, which aims to assess a ship company according to the criteria set out by the ESG/CSR/corporate sustainability approach. Most of the research work under this trend focuses on assessing sustainability reports in the shipping industry. First papers are also being published, detailing the model ESG reporting framework tailored for shipping businesses. It is structured on the basis of the reporting practice of publicly listed shipping companies [57–58], however, it does not take into account the specificity of individual segments of the shipping market. In the maritime industry, knowledge on this subject is particularly lacking in relation to small and medium-sized enterprises [59]. The standards for CSR benchmarking in the shipping industry are still in their early development phases [36]. The need to evaluate CSR/ESG initiatives and to create uniform schemes for such assessment for the maritime industry is highlighted not only by the academic community, but also by the business environment [60].

In our study, the assessment of ferry operators’ attitude toward sustainability was conducted on the basis of ten sustainability categories. Three sources of knowledge were decisive in their selection, as they offered guidelines on how to best understand the concept of corporate sustainability. The first inspiration came from the SDGs which were analysed in terms of feasibility for the maritime industry, the other factor being the considerable openness to selection of important goals, recommended by the International Maritime Organisation (IMO). The second source was the new general guidelines for sustainability reporting and the topics proposed in the European Sustainability Reporting Standards (ESRS). In addition to that, the examined sustainability categories are consistent with corporate sustainability topics which are most often addressed in the literature covering both liner shipping and cruise shipping.

Implementation of strategies and conducting activities contributing to achievement of the SDGs is recommended and supported by the International Maritime Organisation (IMO) – the organisation shaping the framework for the functioning of international shipping. While the IMO recommends commitment to all SDGs, its own legislation focuses on the following SDGs: life below water (SDG_14), climate action (SDG_13), industry, innovation and infrastructure (SDG_9), gender equality (SDG_5), partnerships for the goals (SDG_17) (IMO, 2019). Shipowners themselves refer to different goals. SDG_14 is the most common [61–62], and the remaining goals are selected at the sole discretion of shipowners, using almost the entire range of goals contained in Agenda 2030 [63].

The European Union introduced new sustainability reporting rules. In accordance with the Corporate Sustainability Reporting Directive, the first sustainability reports following the ESRS framework will be published in 2025, and in the subsequent years this obligation will be extended to further groups of companies [25]. What is important, even if ferry companies do not meet the criteria set out by the CSRD and will not be required to report on sustainability, many a time they provide services to larger companies covered by such an obligation. As a transport service provider, they are part of the value chain and their GHG emissions will be relevant to their customers.

The first set of ESRSs, which was used in our study, was adopted by the European Commission in 2023 and provides guidance on information to be made public [64]. The ESRS1 standard covers five environmental, four social and one governance reporting areas. They are structured in general terms and may be applied in every industry. The sector-specific ESRS2 standard, in turn, has not yet been developed [65]. They will be dedicated to nine business sectors, including road transport, but there is no separate standard for shipping. Our research study does not assess the value of information and indices published by the ferry operators, but their approach to disclosure of such information, i.e., openness to sharing information about their own sustainability initiatives. The links between the categories adopted in our study and the respective SDGs and ESRSs are shown in Table 1.

**Table 1:** List of *sustainability categories* (*SCI*) used in the study, and their assignment to ESRS topics and SDGs.

Symbol [SCi]	*Sustainability category* name	ESRS	SDG
SC1	Air pollution emissions	ESRS E2 (Pollution)	SDG11 (Sustainable Cities and Communities) SDG14 (Life below water)
SC2	Greenhouse gas emissions	ESRS E1 (Climate Change)	SDG11 (Sustainable Cities and Communities) SDG13 (Climate Action) SDG14 (Life below water)
SC3	Energy and water consumption	ESRS E1 (Climate Change); ESRS E3 (Water and marine resources)	SDG6 (Clean water and sanitation) SDG7 (Affordable and clean energy)
SC4	Waste management	ESRS E5 (Resource use and circular economy)	SDG12 (Responsible consumption and production) SDG14 (Life below water)
SC5	Food waste mitigation	ESRS E5 (Resource use and circular economy)	SDG2 (Zero hunger) SDG12 (Responsible consumption and production) SDG14 (Life below water)
SC6	Social involvement	ESRS S3 (Affected communities) ESRS S4 (Consumers and end-users)	SDG4 (Quality education) SDG5 (Gender equality) SDG9 (Industry, innovation and infrastructure)
SC7	Application of a code of ethics	ESRS S1 (Own Workforce) ESRS S2 (Workers in the value chain)	SDG5 (Gender equality) SDG10 (Reduced inequalities)
		ESRS G1 (Business Conduct)	
SC8	Cooperation with local partners and suppliers	ESRS S2 (Workers in the value chain)	SDG8 (Decent work and economic growth) SDG12 (Responsible consumption and production) SDG17 (Partnerships for the goals)
		ESRS G1 (Business Conduct)	
SC9	Voyage safety	ESRS S1 (Own Workforce) ESRS S4 (Consumers and end-users)	SDG3 (Good health and well-being) SDG9 (Industry, innovation and infrastructure)
SC10	Support provided to passengers with special needs (e.g., disabilities)	ESRS S4 (Consumers and end-users)	SDG3 (Good health and well-being) SDG9 (Industry, innovation and infrastructure) SDG10 (Reduced inequalities)

Legend for ESRS topics. Green colour represents “Environment”. Cream colour represents “Social”.

In the literature, among the thematic areas related to corporate sustainability of shipping operators in general, there are topics such as environmental protection (e.g., ballast water, oil spills, air pollution and emissions), diversity issues (e.g., gender unequal employment and/or payment issues), and business ethics (e.g., anti-corruption, anti-bribery) [18]. Karagiannis et al. [19], in turn, when identifying materiality issues for the maritime industry, found occupational health and safety, energy management and air emissions to be the most important topics, where innovative technologies and security systems can contribute effectively [19].

In addition to such general issues, there are also more detailed ones, the choice of which depends on the type of shipping segment and business model of a given company. For example, shipowners operating on the cargo market pay close attention to customer expectations and actively address environmental concerns, occupational safety, workers’ rights, and welfare issues [66]. In contrast, passenger shipping companies not only focus on environmental performance – an area shared by all shipping sectors – but also emphasise a broader integration of social and philanthropic initiatives [67]. It is possible to observe not only the diversity of the initiatives taken, understood differently even by actors from the same segment of the shipping market, but also the different motivations behind the decisions made by companies [59].

## 3. Research methodology

### 3.1. Research setting: The baltic and the adriatic

The Baltic Sea and the Adriatic Sea are two important European water areas which differ in terms of location, size, ecological and socio-economic conditions. Sea ferries have become an inextricable marine chain between land routes in most countries. This factor is effective both in the Adriatic and in the Baltic, where ferry lines are connected to the European continent through a thousand islands [68].

The passenger ferry markets in the Baltic Sea and the Adriatic Sea play an important role in the European maritime transport, connecting countries and regions, as well as supporting development of tourism and trade. Both seas are characterised by different operating conditions that affect the operation of ferry services in those waters.

The Baltic Sea is located in Northern Europe. It is situated between Scandinavia and the European continent, and it connects to the North Sea through the Danish Straits. It borders nine countries: Sweden, Finland, Russia, Estonia, Latvia, Lithuania, Poland, Germany, and Denmark, which have well-developed ferry connections. Its surface area amounts to 415 266 km², making it one of the largest inland seas in the world. The Baltic seafloor shows diverse relief features, including numerous depths and shallow areas. It is relatively shallow, with the average depth of about 52 metres, and the maximum depth reaching 459 meters in the Landsort Deep. The Baltic coastline is varied and extends over a length of 8,100 kilometres [53]. In geological terms, the Baltic Sea is young, it is a semi-enclosed and shallow body of brackish water, featuring a unique and sensitive ecosystem. Due to its connection to the North Sea via the Danish Straits, the Baltic Sea is a dynamic ecosystem. This makes it not only an important transport route, but also a high conservation value area requiring special protection.

There are over 50 passenger ports and ferry terminals in the Baltic Sea, which handle regular passenger services. Some ports have a single ferry terminal, while others, such as Tallinn, Helsinki, Turku, and Stockholm, host multiple terminals. The range of ferry services in the Baltic Sea is very diverse, from 30-minute passages on small, 50-person ferry boats to overnight voyages on large ferries that can carry up to 3,000 passengers. Most of the voyages in the northern Baltic Sea are overnight ones, while the southern Baltic is criss-crossed with short-distance ferry connections, e.g., from Poland and Germany to Denmark and Sweden. The study identified 20 ferry operators in the Baltic Sea, offering passenger and car ferry services. The uniqueness of the Baltic area lies in the fact that on some routes, the smallest ferry operators can successfully compete with giants. Additionally, many of the Baltic countries have an insular coast, making passenger ferry connections essential for commuters going to work and shopping for everyday necessities [69].

Table 2 shows the ferry operators in the Baltic Sea and the number of vessels they operate.

**Table 2:** Ferry operators in the Baltic Sea and the number of operated vessels as of 28.05.2024.

Symbol [PBj]	Company name	Headquarters	Number of vessels	Link to website
PB1	Finnlines Plc	Helsinki, Finland	**23**	https://www.finnlines.com/company/about-us/our-fleet/
PB2	Viking Line	Mariehamn, Åland, Finland	**6**	https://www.vikingline.com/the-group/viking-line/vessels/
PB3	Tallink	Tallinn, Estonia	**14**	https://www.tallink.com/fleet
PB4	Eckerö Line	Helsinki, Finland	**2**	https://www.eckeroline.com/company-presentation/eckero-group
PB5	Stena Line	Gothenburg, Sweden	**28**	https://www.stenaline.pl/promy
PB6	DFDS	Copenhagen, Denmark	**80**	https://www.statista.com/statistics/896791/number-of-ships-in-the-fleet-of-dfds-by-type/
PB7	TT-Line	Lübeck-Travemünde, Germany	**10**	https://www.ttline.com/en/ttline/ships/
PB8	Wasaline	Vaasa, Finland	**1**	https://www.wasaline.com/en/our-ferry/
PB9	Unity Line	Szczecin, Poland	**7**	https://www.unityline.pl/promy
PB10	Polferries	Kołobrzeg, Poland	**5**	https://polferries.com/our-ferries/
PB11	Bornholmslinjen	Rønne, Denmark	**3**	https://www.bornholmslinjen.com/about-bornholmslinjen/the-ferries
PB12	Eckerölinjen	Mariehamn, Åland, Finland	**1**	https://www.eckerolinjen.se/en/konferensresor/meeting-on-board
PB13	Destination Gotland	Visby, Gotland, Sweden	**3**	https://www.destinationgotland.se/en/ferry/travel-info/our-ships/
PB14	Scandlines	Copenhagen, Denmark	**7**	https://www.scandlines.com/about-us/our-ferries-and-harbours/
PB15	Żegluga Gdańska	Gdańsk, Poland	**14**	https://www.zegluga.pl/o-nas
PB16	Kołobrzeska Żegluga Pasażerska Sp. z o.o.	Kołobrzeg, Poland	**1**	https://kzp.kolobrzeg.pl/
PB17	TS Laevad OÜ (praamid.ee)	Tallinn, Estonia	**5**	https://www.praamid.ee/en/ferries/leiger/
PB18	Kihnu Veeteed	Sääre, Estonia	**7**	https://www.veeteed.com/#/en/content/fleet
PB19	Tuule Liinid	Muratsi, SAAREMAA ESTONIA	**1**	https://www.tuuleliinid.ee/index.php?option=com_content&view=article&id=209&lang=en
PB20	Saimaa Travel	Lappeenranta, Finland	No data	https://www.saimaatravel.fi/index.html

Source: own elaboration based on a review of ferry operators’ websites.

Most of the routes are served by Ro-Pax ferries which have passenger spaces as well as holds for vehicles and other ro-ro cargoes. Ro-Pax ferries are equipped with passenger cabins, seats on the main deck, shops, restaurants, and entertainment facilities. Vehicles go into the holds and park there for the time of the voyage [12].

The legal regulations applicable in the Baltic Sea Region are stricter than those in other parts of the world, due to its hydrological structure and the marine ecosystem which requires special protection [70]. In 2005, the Baltic Sea area was recognised by the International Maritime Organization (IMO) as a particularly sensitive sea area (PSSA) [71]. The limits of NOx and SOx emissions applicable to the Baltic Sea, as well as the North Sea, are lower than anywhere else in the world. Therefore, shipping companies operating in the area invest in innovative and eco-friendly ferries powered by alternative fuels.

The Adriatic Sea is located between the Apennine Peninsula in the west and the Balkan Peninsula in the east, on the south side it borders the Ionian Sea [72]. It is also a semi-enclosed sea, and it constitutes the largest gulf of the Mediterranean Sea. Its area amounts to ca. 138,600 km², the average depth is 173 m, the maximum depth reaches 1233 meters near Otranto [73]. Although it is one of the shallower seas in the Mediterranean region, it is much deeper than the Baltic Sea [74,75]. The sea is surrounded by five countries: Italy, Croatia, Slovenia, Montenegro and Albania [76]. The environmental conditions found in the Adriatic Sea vary. The northern part of the sea shows lower temperatures and higher salinity waters, which is due to the inflow of river waters and meteorological phenomena [77]. In the southern part of the sea, especially in the Dalmatian region, the waters are warmer, which fosters development of various coral species [78]. The Adriatic Sea is also characterised by complex sea currents that affect its ecosystem [79]. Compared to the Baltic Sea, the Adriatic Sea features higher salinity and warmer waters, which promotes richer biodiversity. The Adriatic is home to numerous species of fish, shellfish, and marine vegetation [76,80]. It is also distinguished by its picturesque coastline, including numerous beaches, bays and islands. The Croatian coast, known for its crystal clear water and charming towns such as Dubrovnik and Split, is particularly popular among tourists. Italy, with its historic cities and beautiful beaches, also attracts crowds of visitors. The Adriatic Sea is surrounded by a variety of landscapes, including mountainous Dalmatia and flat plains in the north. Along the coast, there are numerous islands that attract tourists from all over the world.

In geographical terms, the sea also plays an important role in maritime transport, connecting ports in Italy, Croatia, Slovenia, and other countries. Passenger ports are located along the entire Adriatic and Ionian coasts, with the main freight ports located in the northern Adriatic Sea, where the Mediterranean Sea goes deepest into the European continent, and in the Apulia region in southern Italy, the closest point to Greece and Albania. In the Adriatic/ Ionian region there are about 40 ports that provide ferry services. International ferry ports include: Venice, Ancona, Bari and Brindisi in Italy, Patras and Igoumenitsa in Greece, Split and Dubrovnik in Croatia, Bar in Montenegro and the Albanian port of Durres [72]. Table 3 presents data on the identified ferry operators running their business in the Adriatic Sea.

**Table 3:** Ferry operators in the Adriatic Sea and the number of operated vessels as of 10.11.2024.

S/n	Company name	Headquarters	Number of vessels	Link to website
PA1	Jadrolinija	Rijeka, Croatia	**39**	https://www.jadrolinija.hr/en/fleet-category
PA2	Liberty Lines	Trapani, Italy	**32**	https://www.libertylines.it/en/company/our-fleet/
PA3	Grimaldi Group	Naples, Italy	**46**	https://www.grimaldi.napoli.it/en/fleet/
PA4	Venezia Lines	Poreč, Croatia	**2**	https://www.venezialines.com/about-us/fleet/
PA5	TP Line	Zadar, Croatia	**5**	https://www.tp-line.hr/en/fleet
PA6	Rapska plovidba	Rab, Croatia	**4**	http://www.rapska-plovidba.hr/home_en.html
PA7	GV Line	Dubrovnik, Croatia	**2**	https://www.croatiaferries.com/gv-line/
PA8	Miatrade d.o.o.	Zadar, Croatia	**3**	https://www.miatours.hr/en
PA9	G & V Line Iadera	Zadar, Croatia	**5**	https://www.gv-zadar.com/flota/
PA10	RPZ Vrgada	Vrgada, Croatia	**4**	https://www.vrgada-rpz.hr/rpz-vrgada/flota/
PA11	SNAV	Naples, Italy	**11**	https://www.snav.it/en/la-flotta
PA12	Montenegro Lines	Bar, Montenegro	**2**	https://www.montenegrolines.com/index.php

Source: own elaboration based on a review of ferry operators’ websites

Ferry shipping in the Adriatic Sea plays a key role in passenger and cargo transport on various geographically strategic routes. The ferry transport in the region shows a significant increase in passenger flows and expansion of ferry services that are essential for the tourist industry and local economies [68,72]. Research studies have demonstrated that ferry services constitute an integral part of the transport infrastructure, providing necessary connections between mainland Europe and its islands, thus supporting the tourism sector [72,81]. Integration of ferry services with land transport systems increases accessibility and reduces travel time, making them the preferred choice for many travellers [81,82]. Moreover, ferry routes are not limited to passenger transport only, but also play a significant role in freight transport which is increasingly considered to be more cost-effective compared to land transport [83].

The operational dynamics of ferry transport in the Adriatic are influenced by various factors which create both challenges and opportunities for navigation and route optimisation. The relatively small width of the Adriatic Sea and the changing weather conditions require careful planning and management of ferry routes to ensure safety, increase operational efficiency and quality of service [68,82,84,85]. Furthermore, the environmental implications of ferry operations mean that there is a constant need to minimise carbon emissions and optimise routes in terms of sustainability [82].

### 3.2. Research design

**Fig 1:**
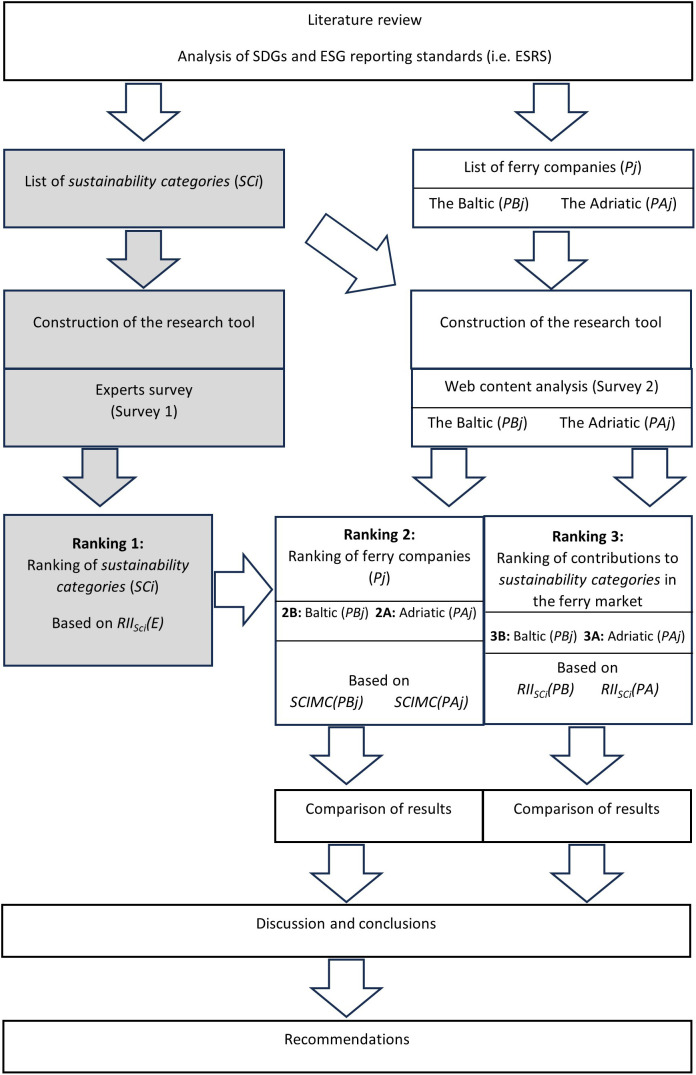
Research procedure.

A diagram of the research procedure is shown in Fig 1. The diagram shows the experts survey (Survey 1) and the resulting ranking of *sustainability categories* (Ranking 1), which are marked in grey. This is the first phase of the research procedure to identify the most important *sustainability categories* for ferry operators [26]. All experts participating in the study provided written informed consent. The experts survey applied the 5-point Likert scale (5 – I definitely agree, 4 – I agree, 3 – I don’t know, 2 – I disagree, 1 – I definitely disagree) to provide answers to questions whether the websites of ferry operators should contain information on *sustainability categories* (SC*i*) presented in Table 1.

The ranking of *sustainability categories* (SC*i*) was developed on the basis of the relative importance index (*RII*) [86], modified as follows:


$${RII}_{SCi}(E)=\frac{\text{5}n_{\text{5}}+\text{4}n_{\text{4}}+\text{3}n_{\text{3}}+\text{2}n_{\text{2}}+\text{1}n_{\text{1}}}{\text{5}\left(n_{\text{5}}+n_{\text{4}}+n_{\text{3}}+n_{\text{2}}+n_{\text{1}}\right)},$$ (1)

where *n*_*1*_*, n*_*2*_*, n*_*3*_*, n*_*4*_ and *n*_*5*_ represent the number of respondents (experts) who selected answers from 1 to 5.

In the decreasing order of the *RII*_*SCi*_*(E)* value (as per Ranking 1), the following weights were assigned to individual SC*i*: 9 – SC9, 8 – SC10, 7 – SC4 and SC7, 6 – SC1, 5 – SC2, 4 – SC6, 3 – SC3, 2 – SC8, 1 – SC5 [26].

The second phase of the research study assessed to what extent the operators published information about their corporate sustainability activities in relation to the examined *sustainability categories*. By way of website content analysis, the ferry companies operating in the Baltic Sea (see Table 2) and in the Adriatic Sea (see Table 3) were examined (Survey 2). The 6-point Likert scale was applied as follows: 0 – No information, 1 – The topic is mentioned, 2 – The topic is mentioned and an extensive explanation included, 3 – One example of action is provided, 4 – More than one example of action is provided, 5 – Information is provided in a separate tab/file/document. The study on the Baltic Sea was carried out by a team of authors from Poland, and the study on the Adriatic Sea – by a team of authors from Croatia. The collection and analysis method complied with the terms and conditions for the source of the data.

Combining the results of Survey 1 and Survey 2, the *SDGs commitment Index in Marketing Communication* (*SCIMC*) was developed [26], which shows the degree of the ferry companies’ engagement in sharing information about their activities assigned to *sustainability categories*. It is calculated as follows:


$$SCIMC(Pj\;)=\frac{\sum_{i=\text{1}}^{\text{1}\text{0}}o_{SCi}(Pj)\cdot w(SCi)}{\sum_{i=\text{1}}^{\text{1}\text{0}}w(SCi)},$$ (2)

where: *o*_*SCi*_*(Pj)* – mark granted to the company website for a particular SCi, *w(SCi)* – weight assigned to that *SCi*.

The ranges of index (2) values and the corresponding degree (level) of the ferry companies’ engagement in informing about their activities related to the examined *sustainability categories* are presented in Table 4.

**Table 4:** *SCIMC(P*_*j*_) value ranges.

$\text{0}\leq SCIMC(P_{j})<\text{2}$	very low
$\text{2}\leq SCIMC(P_{j})<\text{3}$	low
$\text{3}\leq SCIMC(P_{j})<\text{4}$	medium
$\text{4}\leq SCIMC(P_{j})\leq \text{5}$	high

The *SCIMC(P*_*j*_*)* index was applied to establish the ranking of enterprises (Ranking 2).

Separate rankings were obtained as follows:

- Ranking 2B for the enterprises operating in the Baltic Sea, based on *SCIMC(PB*_*j*_*)*,
- Ranking 2A for enterprises operating in the Adriatic Sea, based on *SCIMC(PA*_*j*_*)*.

The next step was a comparative analysis of the rankings received and the relationship between the index value and the number of vessels operated by the surveyed companies. The correlation between the number of vessels and the level of engagement in informing about their actions related to the examined sustainability categories (expressed by the *SCIMC(P*_*j*_*)* index) was determined based on the Pearson linear correlation coefficient (for a significance level 0.05).

In relation to the two marine areas examined, separate rankings of contribution to sustainability categories in the Baltic ferry market (Ranking 3B) and the Adriatic ferry market (Ranking 3A) were also established. To rank sustainability categories based on their presence on the ferry companies’ websites, the relative importance index (RII) [86] was used once more, with the following modifications:


$${RII}_{SCi}(P)=\frac{\text{5}o_{\text{5}}+\text{4}o_{\text{4}}+\text{3}o_{\text{3}}+\text{2}o_{\text{2}}+\text{1}o_{\text{1}}}{\text{5}\left(o_{\text{5}}+o_{\text{4}}+o_{\text{3}}+o_{\text{2}}+o_{\text{1}}+o_{\text{0}}\right)},$$ (3)

where o_0_, o_1_, o_2_, o_3_, o_4_ and o_5_ represent the number of companies awarded a mark from 0 to 5.

Using formula (3), *RII*_*sci*_ *(PB)* and *RII*_*sci*_ *(PA)* indices were determined. Then, a comparative analysis of the obtained results was carried out.

## 4. Results and discussion

**Fig 2:**
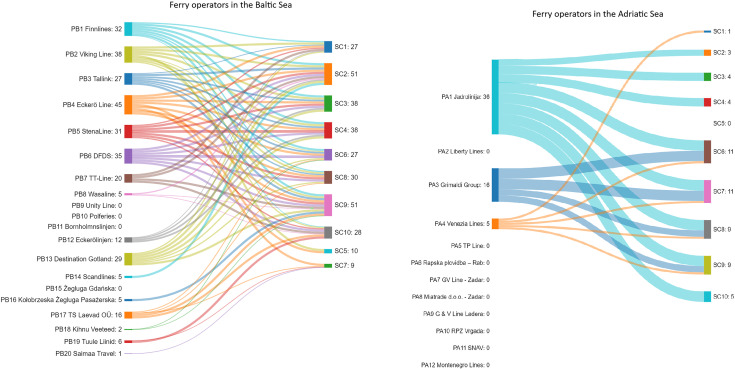
Sustainability communications topics referred to by the ferry operators.

The computed results demonstrate a detailed picture of the approach taken by both ferry operator groups with regard to publishing information on sustainable issues. Prior to studying the detailed results of the indices calculation, the outcome of web content analysis was visualised on the Sankey diagram (Fig 2). The graphical presentation shows which sustainability categories were deployed by the individual companies in their marketing communications. Fig 2 indicates that some of the studied operators scored zero in all the analysed categories. It also exhibits which categories were most often referred to by all the operators in aggregate. What the companies engaged in sustainability communications have in common is a relatively high interest in communicating commitment to voyage safety (SC9).

Table 5 shows the ranking of the analysed Baltic ferry companies, established using the *SCIMC(PB*_*j*_*)* index value – Ranking 2B, whereas Table 6 presents Ranking 2A for the Adriatic ferry companies.

**Table 5:** Ranking of the Baltic ferry companies (Ranking 2B).

Rank	Symbol [*PBj*]	Company name	*SCIMC(PBj)*	Rank	Symbol [*PBj*]	Company name	*SCIMC(PBj)*
1	PB4	Eckerö Line	4.23	11	PB19	Tuule Liinid	0.90
2	PB6	DFDS	3.65	12	PB16	Kołobrzeska Żegluga Pasażerska Sp. z o.o.	0.87
3	PB2	Viking Line	3.58	13	PB8	Wasaline	0.62
4	PB1	Finnlines	3.31	14	PB14	Scandlines	0.48
5	PB5	StenaLine	3.31	15	PB18	Kihnu Veeteed	0.25
6	PB13	Destination Gotland	3.00	16	PB20	Saimaa Travel	0.13
7	PB3	Tallink	2.77	17	PB9	Unity Line	0.00
8	PB7	TT-Line	2.69	18	PB10	Polferries	0.00
9	PB17	TS Laevad OÜ (praamid.ee)	2.13	19	PB11	Bornholmnslinjen	0.00
10	PB12	Eckerölinjen	1.17	20	PB15	Żegluga Gdańska	0.00

**Table 6:** Ranking of the Adriatic ferry companies (Ranking 2A).

Rank	Symbol [*PAj*]	Company name	*SCIMC(PAj)*	Rank	Symbol [*PAj*]	Company name	*SCIMC(PAj)*
1	PA1	Jadrolinija	3.94	7	PA7	GV Line	0.00
2	PA3	Grimaldi Group	1.69	8	PA8	Miatrade d.o.o.	0.00
3	PA4	Venezia Lines	0.54	9	PA9	G & V Line Iadera	0.00
4	PA2	Liberty Lines	0.00	10	PA10	RPZ Vrgada	0.00
5	PA5	TP Line	0.00	11	PA11	SNAV	0.00
6	PA6	Rapska plovidba	0.00	12	PA12	Montenegro Lines	0.00

Based on the results obtained, it can be concluded that among the surveyed ferry companies it was Eckerö Line operating in the Baltic Sea area that was characterised by the highest degree of engagement in informing about their activities related to the examined *sustainability categories*. Nine out of ten categories received the highest ratings. This company’s website lacked only information about support provided to passengers with special needs (SC10). A slightly lower index value was obtained by the Jardolinija company operating in the Adriatic Sea. However, this figure falls within the medium level of activity. No information was found with regard to air pollution emissions (SC1) or food waste mitigation (SC5). The latter of these categories was considered by the experts as the least important. The same, medium level of engagement as shown by Jardolinija was found for five Baltic companies, with the index values ranging from 3.00 to 3.65. All the maximum subscores earned by these companies’ websites were obtained for greenhouse gas emissions (SC2), energy and water consumption (SC3), and waste management (SC4). At the same time, none of these companies published information about application of a code of ethics (SC7). The three Baltic companies that showed a low level of engagement received the maximum rating for publishing voyage safety information (SC9). However, they did not disclose any information about any food waste mitigation activities (SC5). As many as twenty-two (eleven Baltic and eleven Adriatic) companies demonstrated a very low level of engagement in informing about their activities related to the examined *sustainability categories* (i.e., the index value was lower than 2.0). It is worth noting one of these companies – Grimaldi Group, which ranked second among entities operating in the Adriatic Sea. It received the highest subscores in two categories, namely social involvement (SC6) and application of a code of ethics (SC7). Four Baltic and nine Adriatic companies achieved zero index values – on their websites they did not present any information regarding the analysed *sustainability categories*.

**Fig 3:**
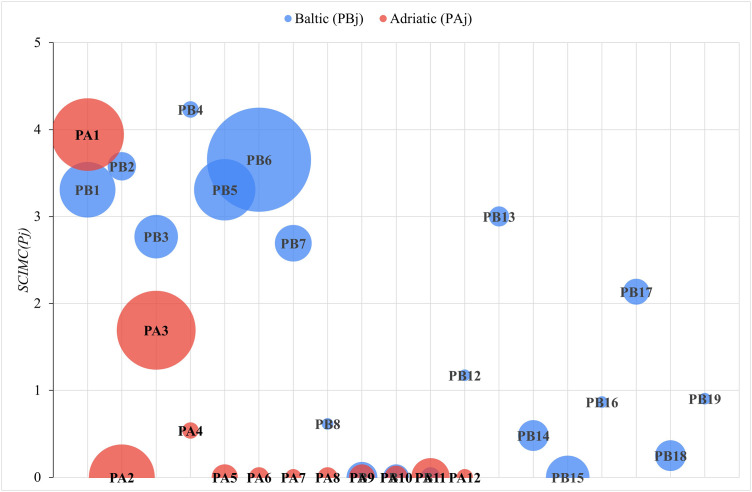
The studied ferry companies displayed as per the *SCIMC (P*_*j*_) index level, and the number of vessels (size of the bubble).

**Fig 4:**
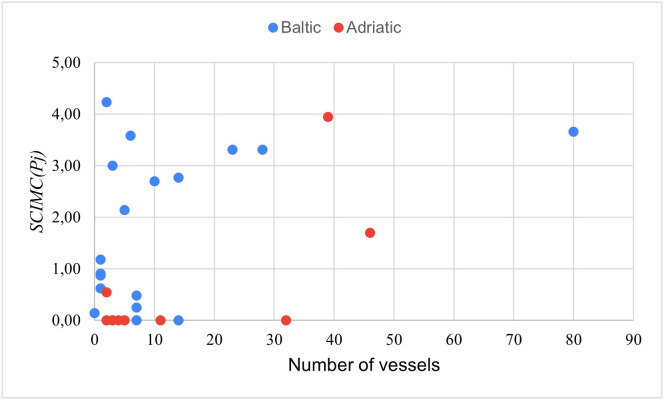
Dispersion of the *SCIMC (P*_*j*_) index values in relation to the number of vessels operated by the studied ferry companies.

Fig 3 shows the *SCIMC(P*_*j*_*)* index values displayed in Tables 5 and 6, taking into account the number of vessels operated by the given company (Tables 2 and 3). Fig 4, in turn, is a graph showing the dispersion of the index values in relation to the number of vessels operated by the companies.

As for the companies operating in the Baltic Sea, no significant correlation was found between the number of vessels operated and the degree of engagement in informing about any measures taken by them in relation to the *sustainability categories* examined. Eckerö Line, as the leader of Ranking 2B, operated only 2 ferries. In contrast, the group of companies characterised by a medium level of engagement is quite diverse: the number of vessels varied from 3 to 80. At the same time, it is a group whose total number of vessels held (140) accounted for 64% of the total number of vessels held by the surveyed Baltic companies (218). The enterprises with a very low level of engagement usually had several ships, as many as four of them – only one vessel. The exception is Żegluga Gdańska with 14 ferries.

At the same time, a significant moderate correlation (correlation coefficient of 0.72) was observed between the number of vessels at the disposal of the company operating in the Adriatic Sea and the degree of its engagement in informing about its activities related to the categories examined. Unlike the Baltic companies, the leader of Ranking 2A is one of the three enterprises with the highest number of vessels (39). Jadrolinija is an enterprise that mainly serves passengers on local routes connecting ports in Croatian islands. The companies that published no information about the examined categories were mainly those with the number of ferries ranging from 2 to 11. The exception in this group is Liberty Lines with 32 vessels.

**Fig 5:**
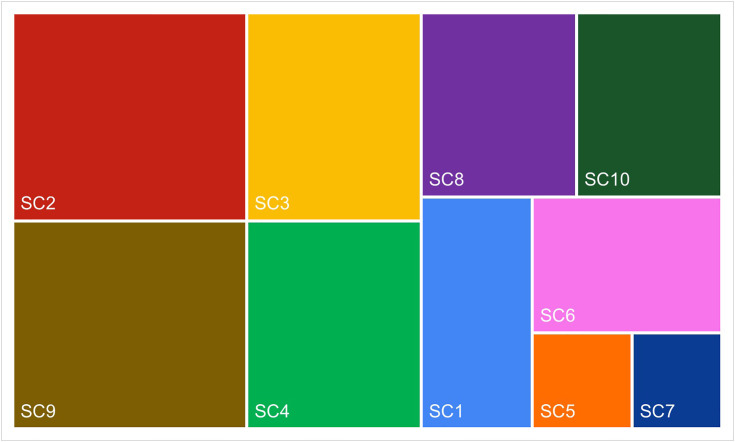
Contributions to sustainability categories by the Baltic ferry market.

**Fig 6:**
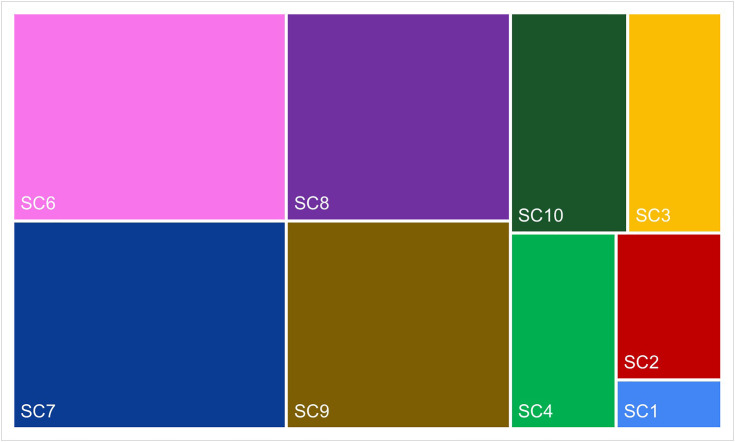
Contributions to sustainability categories by the Adriatic ferry market.

**Fig 7:**
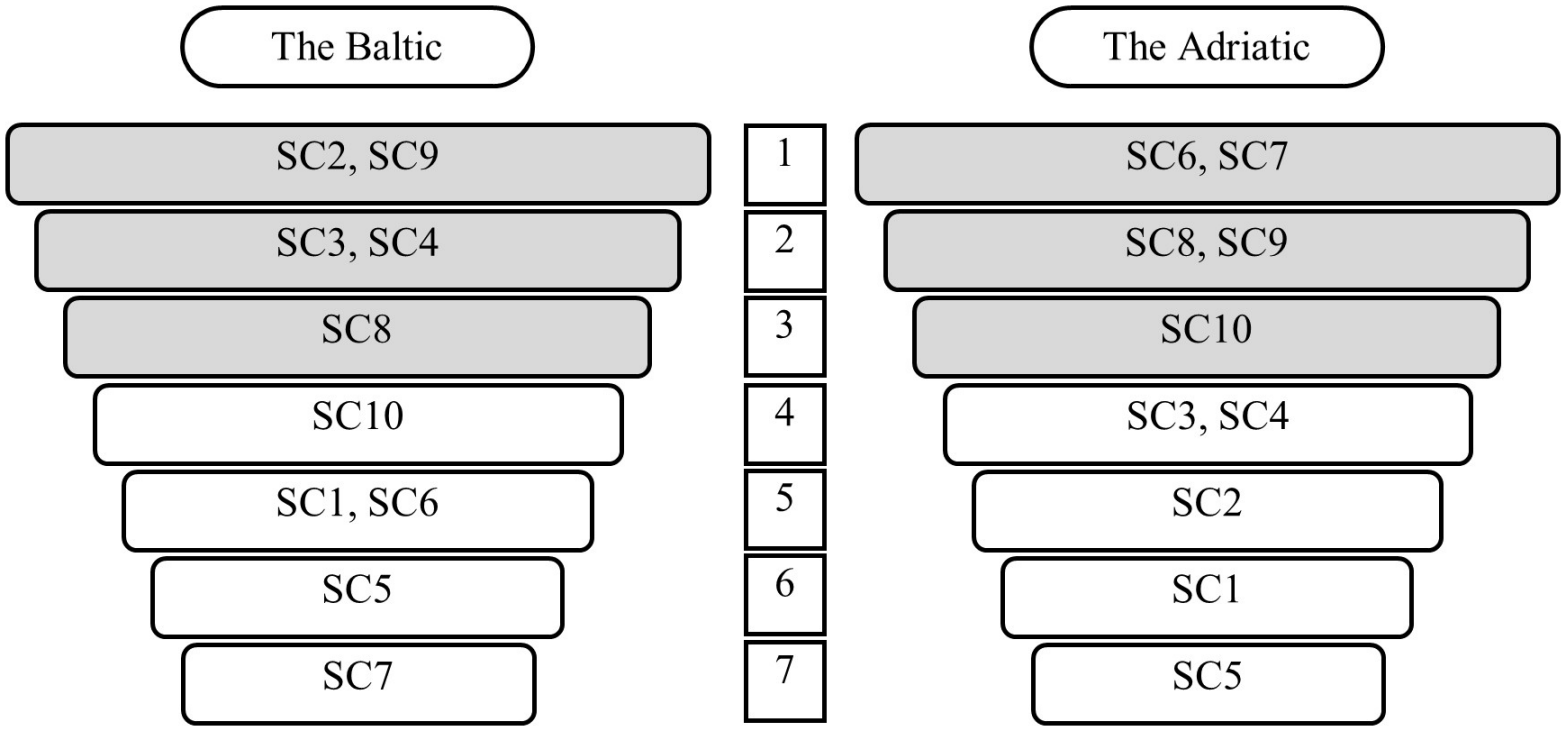
Rankings of contributions to sustainability categories by the Baltic and the Adriatic ferry market (Ranking 3B and Ranking 3A).

Subsequently, calculations were carried out for the entire Baltic and Adriatic ferry markets. Contributions to sustainability categories by the overall Baltic ferry market, according to the *RII*_*sci*_*(PB)* index, are presented in Fig 5. Contributions to sustainability categories by the overall Adriatic ferry market, according to the *RII*_*sci*_ *(PA*) index, are shown in Fig 6. Fig 7, in turn, shows the rankings of contributions to sustainability categories by the Baltic and Adriatic ferry market (Ranking 3B and Ranking 3A).

According to the rankings obtained, certain differences in the importance of individual *sustainability categories* were observed in the surveyed ferry markets. Looking at the top three in the ranking, only two categories came up twice, i.e., voyage safety (SC9) and cooperation with local partners and suppliers (SC8). The former is also the area indicated by experts (in Survey 1) as the most important, while the latter as the penultimate in Ranking 1 [26]. The Baltic companies also attached great importance to greenhouse gas emissions (SC2). In the case of the Adriatic companies, social involvement (SC6) and application of a code of ethics (SC7) came on top. Both of these categories fell within the lower part of the Baltic market ranking, and SC7 even turned out to be the last. According to the experts, information on food waste mitigation (SC5) was the least relevant, which was also confirmed in the study of both markets. The Adriatic ferry companies did not even mention it.

## 5. Conclusions

As a result of the conducted research, it was possible to answer both research questions formulated in the initial phase of the research process.

The first research question (RQ1) was aimed at identifying which of the ferry operators functioning in the examined marine areas were the most engaged in sharing information about sustainability issues. In the research process, the original index (SCIMC) was applied to assess the degree of engagement of the ferry companies in sharing information about their activities assigned to sustainability categories. It was found that the operators functioning in the Baltic area showed much greater engagement in informing about sustainability issues, compared to those operating in the Adriatic area, even though in both areas the predominant level of engagement was very low (SCIMC < 2). In the Baltic region, such engagement was shown by 55% of the surveyed companies, whereas among the Adriatic operators a very low level of engagement was demonstrated by 92% of them. Only one company – Jadrolinija – operating in the Adriatic region achieved an index indicating that their commitment to sustainability issues was medium. Among the Baltic companies, this index value was obtained by 25% of the surveyed operators. There was only one operator in the Baltic area (Eckerö Line) that managed to achieve the SCIMC index value reflecting a high level of commitment to informing about sustainability issues.

By organising the operators in the descending order of the SCIMC index values, it can be concluded that among the five companies for which the SCIMC index reached the highest value, there was only one operating in the Adriatic region – the aforementioned Jadrolinija. The descending order is as follows: Eckerö Line, Jadrolinija, DFDS, Viking Line, Finnlines. It should be noted that all of the top five companies that operate in the Baltic Sea are Scandinavian companies, including as many as three Finnish enterprises. This may indicate that in the Nordic countries sharing information about any activities taken with regard to sustainable development is an accepted standard. Given the high public awareness of the importance of achieving sustainability goals, this can affect consumer choices regarding the choice of operator and consequently have an impact on the financial performance of the company. Perhaps the social awareness in the countries located around the studied seas varies, and hence such substantial differences in the adopted strategy of sharing information about sustainability issues. It should be emphasised that the conducted research study does not allow to determine the actual degree of implementation of any SDGs or CSR initiatives undertaken by the surveyed companies. It is only possible to evaluate the communications provided on the operators’ websites, addressed to individual customers and business partners.

Another reason for the higher SCIMC index levels achieved by the Baltic operators in relation to those from the Adriatic may be the customer profile. Freight transport accounts for a significant share of the Baltic operators’ business, while in the Adriatic the passenger transport prevails. The new regulations on ESG reporting may increase the entrepreneurs’ interest in activities undertaken by ferry operators in this respect. This may be the reason why they place this content on their websites which often constitute the first source of information for potential customers.

The findings from the conducted research also allowed us to answer RQ2 and indicate similarities as well as dissimilarities in sharing information about sustainability issues by the respective groups of studied ferry operators. In addition to the fact that the Adriatic operators much more seldom informed the public about their sustainability-related activities, it was also noted that even if their websites contained communications concerning sustainable issues, they usually concerned different activities than those communicated by the Baltic operators. The Adriatic companies most often provided information about their activities assigned to social involvement (SC6) and application of a code of ethics (SC7) categories. Information about these categories rarely appeared on the Baltic operators’ websites (they fell within the lower part of the ranking). In contrast, the Baltic operators reported on activities assigned to the following categories: greenhouse gas emissions (SC2), energy and water consumption (SC3) and waste management (SC4), which the Adriatic operators rarely reported.

Again, the reason for the differences may be the profile of customers to whom the website communications were addressed. In the case of the Adriatic operators, the categories appearing on the websites may be of more interest to individual customers, while the information provided by the Baltic operators may be more likely to be of interest to business customers, such as forwarding companies.

The identified similarities concerned information about activities that could fall under the following categories: voyage safety (SC9) and cooperation with local partners and suppliers (SC8), which most often appeared on the websites of both studied ferry operator groups. These categories can be considered important not only in the context of SDGs implementation, but also in building relationships with members of the environment, which is essential for gaining customers.

Based on the findings of the research study carried out, in the case of the Baltic companies no correlation was found between sharing information about sustainability issues and the number of vessels held by the operator, whereas for the Adriatic operators this relationship was apparent. Jadrolinija, whose SCIMC index reached the highest value among all the operators in that area, is also the largest ferry operator in the Adriatic. Offering comprehensive information about sustainability issues on the company’s website may set certain trends for the smaller operators in the Adriatic.

To sum up, this article presents research findings that are unique due to the scale of the research area, concerning provision of information about sustainability-related activities by ferry operators functioning in two different seas: the Baltic Sea and the Adriatic Sea.

The outcome of the study presented herein may have a theoretical as well as practical value. In theoretical terms, this paper is the first comparative research study related to reporting on sustainable issues, focusing on two important marine areas – the Baltic Sea and the Adriatic Sea. The study fills a research gap, as the topic of communications made by ferry companies with regard to their attitude to sustainability issues is very rarely addressed in the academic literature.

In practical terms, this article presents research findings that can be used by actors of the supply side of the ferry market, e.g., to implement good practices in their communications. The underlying reason for this is that sharing information about sustainable issues constitutes an important element of communication strategies pursued by some companies.

The limitation of the study presented herein is that it focused only on actors of the supply side of the market, while examining representatives of the demand side could provide us with a broader perspective. It would make it possible to assess the reasons for the differences between the adopted standards of information sharing for companies operating in both of the seas. It would be possible to determine whether the widespread lack of information on sustainable issues among the Adriatic operators was a conscious decision and can be attributed, e.g., to the differences in the social awareness in the Baltic and Adriatic countries and the desire to adapt the website content to the users’ expectations and kind of information the customers actually expected.

## Supporting information

S1 File Survey 1 data. (XLSX)

S2 File Survey 2 data. (XLSX)

## References

[1] Choudhary P, G VS, Khade M, Savant S, Musale A, G RKK, et al. Empowering blue economy: From underrated ecosystem to sustainable industry. J Environ Manage. 2021;291:112697. doi: 10.1016/j.jenvman.2021.112697 33934021

[2] Di Vaio A, Varriale L. Sustainable development goals in the cruise industry: the contribution of sustainability disclosure. In: Nautical and maritime culture, from the past to the future. IOS Press; 2019. 142–8.

[3] Gavalas D, Syriopoulos T. Applying the corporate social responsibility to the shipping industry. Int J Acct Finan Rep. 2018;8(1):155. doi: 10.5296/ijafr.v8i1.12701

[4] Belz FM, Binder JK. Sustainable entrepreneurship: a convergent process model. Bus Strat Env. 2015;26(1):1–17. doi: 10.1002/bse.1887

[5] Fortune Business Insight. Passenger ferries market size, share & industry analysis, by booking type (private and commercial), by type (multihull and monohull), and regional forecast, 2024-2032. 2024. https://www.fortunebusinessinsights.com/passenger-ferries-market-103655

[6] Interferry. Ferry industry facts. Accessed 2024 August 20. https://interferry.com/ferry-industry-facts/

[7] Mannarini G, Carelli L, Salhi A. EU-MRV: an analysis of 2018’s Ro-Pax CO 2 data. In: 2020 21st IEEE International Conference on Mobile Data Management (MDM), 2020. 287–92.

[8] HELCOM. Emissions from Baltic Sea shipping in 2023. 2024. https://helcom.fi/wp-content/uploads/2025/02/Emissions-from-Baltic-Sea-shipping-in-2023_.pdf

[9] EEA. How climate change impacts marine life. European environment agency. 2023. Accessed 2025 October 28. https://www.eea.europa.eu/en/analysis/publications/how-climate-change-impacts-marine-life

[10] Ferry companies in Croatia, Accessed on 2024 March 18. 2024. https://www.ferryhopper.com/pl/ferry-operators?country=HR

[11] Direct feries. Discover our Ferry Companies, Accessed 2024 March 20. https://www.directferries.co.uk/operators.htm

[12] Kaczerska D, Kaczerski K. Współczesne tendencje w promowej żegludze pasażerskiej. Transport Miejski i Regionalny. 2021.

[13] Mańkowska M. Kierunki zmian w strukturze bałtyckiej floty promowej i ich wpływ na segment przewozów pasażerskich. Marketing i Zarządzanie. 2016;42(1):65–78.

[14] Kotowska I. Żegluga morska bliskiego zasięgu w świetle idei zrównoważonego rozwoju transportu. Wydawnictwo Naukowe Akademii Morskiej; 2014.

[15] Kotowska I, Łapko A, Jendryczka V. Identifying and hierarchizing factors that affect the choice of transport routes in land ferry transport chains. ASPAL. 2024;23(2):243–58. doi: 10.31648/aspal.9523

[16] Di Vaio A, Varriale L, Lekakou M, Stefanidaki E. Cruise and container shipping companies: a comparative analysis of sustainable development goals through environmental sustainability disclosure. Maritime Pol Manag. 2020;48(2):184–212. doi: 10.1080/03088839.2020.1754480

[17] Froholdt LL. The perception of corporate social responsibility in the maritime industry. In: WMU studies in maritime affairs. Springer International Publishing; 2018. 5–23. doi: 10.1007/978-3-319-69143-5_2

[18] Akkan E. CSR activities in maritime and shipping industries. In: Advances in business strategy and competitive advantage. IGI Global; 2019. 276–98. doi: 10.4018/978-1-5225-7715-7.ch016

[19] Karagiannis I, Vouros P, Sioutas N, Evangelinos K. Mapping the maritime CSR agenda: a cross-sectoral materiality analysis of sustainability reporting. J Clean Prod. 2022;338:130139. doi: 10.1016/j.jclepro.2021.130139

[20] Zhou Y, Wang X, Yuen KF. Sustainability disclosure for container shipping: a text-mining approach. Transp Pol. 2021;110:465–77. doi: 10.1016/j.tranpol.2021.06.020

[21] Wang X, Yuen KF, Wong YD, Li KX. How can the maritime industry meet Sustainable Development Goals? An analysis of sustainability reports from the social entrepreneurship perspective. Transport Res Part D: Transp Environ. 2020;78:102173. doi: 10.1016/j.trd.2019.11.002

[22] Kizielewicz J. The commitment of global cruise shipping companies to sustainable development. Curr Issues Tourism. 2023;27(11):1683–99. doi: 10.1080/13683500.2023.2217351

[23] Di Vaio A, Van Engelenhoven E, Raimo N, Garofalo A. Strategic carbon disclosure and accountable efficiency: reporting shipping industry scope 3 emissions. Business Strat Environ. 2025. doi: 10.1002/bse.70210

[24] Rehmatulla N, Schwarz K, Habibi Nameghi F. Revisiting the role of private voluntary standards and initiatives in decarbonising shipping. Marine Policy. 2025;179:106707. doi: 10.1016/j.marpol.2025.106707

[25] European Parliament Council. Directive (EU) 2022/2464 of the European Parliament and of the Council of 14 December 2022 amending Regulation (EU) No 537/2014, Directive 2004/109/EC, Directive 2006/43/EC and Directive 2013/34/EU, as regards corporate sustainability reporting. 2022. https://eur-lex.europa.eu/legal-content/EN/TXT/?uri=CELEX:32022L2464

[26] Wagner N, Łapko A, Hącia E, Strulak-Wójcikiewicz R. Commitment to sustainable development goals in marketing communications of ferry companies. PLoS One. 2024;19(12):e0312767. doi: 10.1371/journal.pone.0312767 39621671 PMC11611085

[27] Derqui B. Towards sustainable development: evolution of corporate sustainability in multinational firms. Corp Soc Responsib Env. 2020;27(6):2712–23. doi: 10.1002/csr.1995

[28] Urdan MS, Luoma P. Designing effective sustainability assignments: how and why definitions of sustainability impact assignments and learning outcomes. J Manag Edu. 2020;44(6):794–821. doi: 10.1177/1052562920946798

[29] Pazienza M, de Jong M, Schoenmaker D. Clarifying the concept of corporate sustainability and providing convergence for its definition. Sustainability. 2022;14(13):7838. doi: 10.3390/su14137838

[30] European Commission. A renewed EU strategy 2011-14 for corporate social responsibility. 2011. https://eur-lex.europa.eu/legal-content/EN/TXT/PDF/?uri=CELEX:52011DC0681

[31] Chwiłkowska-Kubala A, Cyfert S, Malewska K, Mierzejewska K, Szumowski W, Prause G. What drives organizational agility in energy sector companies? The role of strategic CSR initiatives and the dimensions of proactive CSR. Sustain Fut. 2023;6:100133. doi: 10.1016/j.sftr.2023.100133

[32] Muhammad A, Alhamzah A. Integrating corporate social responsibility into business strategy: creating sustainable value. Involvem Int J Business. 2024;1(1):29–42. doi: 10.62569/iijb.v1i1.5

[33] Al-Shammari MA, Banerjee SN, Rasheed AA. Corporate social responsibility and firm performance: a theory of dual responsibility. Manang Decision. 2021;60(6):1513–40. doi: 10.1108/md-12-2020-1584

[34] Ang R, Shao Z, Liu C, Yang C, Zheng Q. The relationship between CSR and financial performance and the moderating effect of ownership structure: evidence from Chinese heavily polluting listed enterprises. Sustain Prod Consump. 2022;30:117–29. doi: 10.1016/j.spc.2021.11.030

[35] Vishwanathan P, van Oosterhout H (J. ), Heugens PPMAR, Duran P, van Essen M. Strategic CSR: a concept building meta‐analysis. J Manag Stud. 2019;57(2):314–50. doi: 10.1111/joms.12514

[36] Yuen KF, Lim JM. Barriers to the implementation of strategic corporate social responsibility in shipping. The Asian J Shipp Logist. 2016;32(1):49–57. doi: 10.1016/j.ajsl.2016.03.006

[37] Fan L, Wang M, Huang S, Li Z. Fleet sustainability of the liner market—from the perspective of corporate social responsibility. Maritime Pol Manag. 2024;52(2):173–89. doi: 10.1080/03088839.2024.2343310

[38] Drobetz W, Merikas A, Merika A, Tsionas MG. Corporate social responsibility disclosure: the case of international shipping. Transport Res Part E: Logist Transport Rev. 2014;71:18–44. doi: 10.1016/j.tre.2014.08.006

[39] Yuen KF, Thai VV, Wong YD. Corporate social responsibility and classical competitive strategies of maritime transport firms: a contingency-fit perspective. Transp Res Part A: Pol Pract. 2017;98:1–13. doi: 10.1016/j.tra.2017.01.020

[40] Kaźmierczak M. A literature review on the difference between CSR and ESG. Scit Papers Silesian Univ Tech. 2022;2022(162):275–89. doi: 10.29119/1641-3466.2022.162.16

[41] Yoon B, Chung Y. The effects of corporate social responsibility on firm performance: A stakeholder approach. Journal of Hospitality and Tourism Management. 2018;37:89–96. doi: 10.1016/j.jhtm.2018.10.005

[42] Fernando S, Lawrence S. A theoretical framework for CSR practices: integrating legitimacy theory, stakeholder theory and institutional theory. J Theoret Account Res. 2014;10(1):149–78.

[43] Villamor GB, Wallace L. Corporate social responsibility: current state and future opportunities in the forest sector. Corp Soc Responsib Env. 2024;31(4):3194–209. doi: 10.1002/csr.2743

[44] Yuen KF, Wang X, Wong YD, Zhou Q. Antecedents and outcomes of sustainable shipping practices: The integration of stakeholder and behavioural theories. Transp Res Part E: Logist Transport Rev. 2017;108:18–35. doi: 10.1016/j.tre.2017.10.002

[45] Gilal FG, Paul J, Gilal NG, Gilal RG. Strategic CSR‐brand fit and customers’ brand passion: Theoretical extension and analysis. Psychology and Marketing. 2021;38(5):759–73. doi: 10.1002/mar.21464

[46] Ahn J, Kwon J. CSR perception and revisit intention: the roles of trust and commitment. J Hospital Tourism Insight. 2020;3(5):607–23. doi: 10.1108/jhti-02-2020-0022

[47] Wagner N. Bibliometric analysis of research on green shipping practices. In: Springer proceedings in business and economics. Springer International Publishing; 2019. 323–32. doi: 10.1007/978-3-030-17743-0_27

[48] Di Vaio A, Hassan R, D’Amore G, Dello Strologo A. Digital technologies for sustainable waste management on-board ships: an analysis of best practices from the cruise industry. IEEE Trans Eng Manage. 2024;71:12715–28. doi: 10.1109/tem.2022.3197241

[49] Wagner N, Wiśnicki B. The importance of emerging technologies to the increasing of corporate sustainability in shipping companies. Sustainability. 2022;14(19):12475. doi: 10.3390/su141912475

[50] Łapko A. Age simulation suits in education and training of staff for the nautical tourism sector. Sustainability. 2023;15(4):3803. doi: 10.3390/su15043803

[51] Zhou Y, Li X, Wang X, Yuen KF. Intelligent container shipping sustainability disclosure via stakeholder sentiment views on social media. Marine Policy. 2022;135:104853. doi: 10.1016/j.marpol.2021.104853

[52] Voorveld HAM. Brand communication in social media: a research agenda. J Advert. 2019;48(1):14–26. doi: 10.1080/00913367.2019.1588808

[53] Kierski K. Gdzie jest Bałtyk? Poznaj dokładne położenie największego morza Europy. Accessed 2024 November 11. https://swjangdansk.pl/gdzie-jest-baltyk-poznaj-dokladne-polozenie-najwiekszego-morza-europy

[54] Gibson M, Murphy AJ, Pazouki K. Evaluation of environmental performance indices for ships. Transp Res Part D: Transp Environ. 2019;73:152–61. doi: 10.1016/j.trd.2019.07.002

[55] Christodoulou A, Cullinane K (2021). Potential for, and drivers of, private voluntary initiatives for the decarbonisation of short sea shipping: evidence from a Swedish ferry line. Maritime Econ Logist. 23(4), 632–54. doi: 10.1057/s41278-020-00160-9

[56] Alamoush AS, Ölçer AI, Ballini F. Ports’ role in shipping decarbonisation: a common port incentive scheme for shipping greenhouse gas emissions reduction. Cleaner Logist Supply Chain. 2022;3:100021. doi: 10.1016/j.clscn.2021.100021

[57] Tsatsaronis M, Syriopoulos T, Karamperidis S, Boura G. Shipping-specific ESG rating and reporting framework. Maritime Pol Manag. 2024;51(5):698–716. doi: 10.1080/03088839.2024.2342730

[58] Tsatsaronis M, Syriopoulos T, Karamperidis S, Boura G, Dimopoulos A. ESG reporting quality assessment in listed companies of maritime sector. Default J. 2023.

[59] Fjørtoft BE, Grimstad SMF, Glavee-Geo R. Motivations for CSR in the Norwegian maritime cluster: stakeholder perspectives and policy implications. Maritime Pol Manag. 2020;47(8):1010–26. doi: 10.1080/03088839.2020.1735654

[60] Makris E, Stergiou M. ESG in the shipping sector. The role of ESG in the evaluation of shipping companies. 2019. https://www2.deloitte.com/gr/en/pages/consumer-business/articles/esg-in-the-shipping-sector.html

[61] Neumann B, Ott K, Kenchington R. Strong sustainability in coastal areas: a conceptual interpretation of SDG 14. Sustain Sci. 2017;12(6):1019–35. doi: 10.1007/s11625-017-0472-y 30147766 PMC6086248

[62] Ntona M, Morgera E. Connecting SDG 14 with the other sustainable development goals through marine spatial planning. Marine Pol. 2018;93:214–22. doi: 10.1016/j.marpol.2017.06.020

[63] Xue Y, Lai K. Responsible shipping for sustainable development: adoption and performance value. Transp Pol. 2023;130:89–99. doi: 10.1016/j.tranpol.2022.11.007

[64] EU. Commission Delegated Regulation (EU) 2023/2772 of 31 July 2023 supplementing Directive 2013/34/EU of the European Parliament and of the Council as regards sustainability reporting standards. 2023. https://eur-lex.europa.eu/legal-content/en/ALL/?uri=CELEX:32023R2772

[65] EFRAG. EFRAG. Accessed 2024 September 8. https://www.efrag.org/en/sustainability-reporting/esrs-workstreams/sectorspecific-esrs

[66] Tang L, Gekara V. The importance of customer expectations: an analysis of CSR in container shipping. J Bus Ethics. 2018;165(3):383–93. doi: 10.1007/s10551-018-4062-4

[67] Geerts M, Dooms M. An analysis of the CSR portfolio of cruise shipping lines. Res Transp Busin Manag. 2022;45:100615. doi: 10.1016/j.rtbm.2020.100615

[68] Krile S, Maiorov N, Fetisov V. Research of marine ferry systems based on discretization pf processes and simulation ferry market based on CIRCOS intensity graph. Naše more. 2020;67(1):45–52. doi: 10.17818/nm/2020/1.7

[69] Megalist. Megalist of Baltic sea ferry operators 2023. 2023. Accessed 2024 May 28. https://www.ferryscan.com/info/megalist-of-baltic-sea-ferry-operators

[70] Nasz Bałtyk – hydrologia. Accessed 2023 September 13. https://naszbaltyk.pl/hydrologia

[71] Pyć D. Instrumenty prawnej ochrony Morza Bałtyckiego jako obszaru morskiego o szczególnej wrażliwości. GSP. 2023;(4(61)/2023):137–58. doi: 10.26881/gsp.2023.4.08

[72] Zanne M, Twrdy E, Zlak B, Stojaković M. Development possibilities for ferry transport in Adriatic-Ionian region. Ekonomiczne Problemy Usług. 2016;124:39–52. doi: 10.18276/epu.2016.124-04

[73] Bonacci O, Žaknić-Ćatović A, Roje-Bonacci T. Prominent increase in air temperatures on two small mediterranean islands, Lastovo and Lošinj, Since 1998 and its effect on the frequency of extreme droughts. Water. 2024;16(22):3175. doi: 10.3390/w16223175

[74] Loia M, Nicoletti L, La Porta B. First record of genus Paramphitrite (Polychaeta: Terebellidae) in Mediterranean Sea. Mar Biodivers Rec. 2017;10(1). doi: 10.1186/s41200-017-0113-2

[75] Zonn IS, Kostianoy AG, Semenov AV, Joksimović A, Đurović M. Adriatic Sea. In: Encyclopedia of Seas. Springer International Publishing; 2021. 9–14. doi: 10.1007/978-3-030-50032-0_15

[76] Costantino G, Mastrototaro F, Tursi A, Torchia G, Pititto F, Salerno G, et al. Distribution and bio-ecological features of Posidonia oceanicameadows along the coasts of the southern Adriatic and northern Ionian Seas. Chem Ecol. 2010;26(sup1):91–104. doi: 10.1080/02757541003705484

[77] Novosel M, Požar‐Domac A, Pasarić M. Diversity and distribution of the bryozoa along underwater cliffs in the Adriatic Sea with special reference to thermal regime. Marine Ecol. 2004;25(2):155–70. doi: 10.1111/j.1439-0485.2004.00022.x

[78] Grubelić I, Antolić B, Despalatović M, Grbec B, Paklar GB. Effect of climatic fluctuations on the distribution of warm-water coral Astroides calycularis in the Adriatic Sea: new records and review. J Mar Biol Ass. 2004;84(3):599–602. doi: 10.1017/s0025315404009609h

[79] Pulcinella J, Bonanomi S, Colombelli A, Fortuna CM, Moro F, Lucchetti A, et al. Bycatch of loggerhead turtle (Caretta caretta) in the Italian Adriatic midwater pair trawl fishery. Front Mar Sci. 2019;6. doi: 10.3389/fmars.2019.00365

[80] Zorica B, Vilibić I, Keč VČ, Šepić J. Environmental conditions conducive to anchovy (Engraulis encrasicolus) spawning in the Adriatic Sea. Fisheries Oceanography. 2012;22(1):32–40. doi: 10.1111/fog.12002

[81] Urbanyi-Popiolek I. Management of the ferry shipping and ferry ports in regional transport systems between continental Europe and the Nordic countries. ERSJ. 2021;XXIV(Special Issue 4):524–34. doi: 10.35808/ersj/2786

[82] Mannarini G, Carelli L, Orović J, Martinkus C, Coppini G. Towards least-CO2 ferry routes in the Adriatic Sea. J Marine Sci Eng. 2021;9(2):115. doi: 10.3390/jmse9020115

[83] Nikolina BP, Jian Hua Z. Technical and scale efficiency analysis of 25 nort mediterranean ports: a data envelopment analysis approach. Int J Econ Finan. 2020;12(3):55. doi: 10.5539/ijef.v12n3p55

[84] Parunov J, Ćorak M, Pensa M. Wave height statistics for seakeeping assessment of ships in the Adriatic Sea. Ocean Engineering. 2011;38(11–12):1323–30. doi: 10.1016/j.oceaneng.2011.06.001

[85] Maiorov N, Fetisov V, Krile S, Miskovic D. Simulation of the route network and ferry traffic intensity based on the process of discretization and circos plot intensity diagram. Transport Problems. 2019;14(4):21–30. doi: 10.20858/tp.2019.14.4.2

[86] Alaghbari W, Al-Sakkaf AA, Sultan B. Factors affecting construction labour productivity in Yemen. Inter J Constr Manag. 2017;19(1):79–91. doi: 10.1080/15623599.2017.1382091

